# Microstructure, Mechanical Properties, and Residual Stress Distribution of AISI 316L Stainless Steel Part Fabricated by Laser Metal Deposition

**DOI:** 10.1155/2020/4831798

**Published:** 2020-07-25

**Authors:** Jiayang Gu, Ruifeng Li, Yi Qiu, Hangyu Yue, Bin Liu, Heng Gu

**Affiliations:** ^1^Marine Equipment and Technology Institute, Jiangsu University of Science and Technology, Zhenjiang, Jiangsu 212003, China; ^2^School of Materials Science and Engineering, Jiangsu University of Science and Technology, Zhenjiang, Jiangsu 212003, China; ^3^Cardiff School of Engineering, Cardiff University, Cardiff CF24 3AA, UK

## Abstract

In this paper, AISI 316L stainless steel part is obtained by laser metal deposition additive manufacturing method. The microstructure of the part was observed and analyzed by an optical microscope. The tensile mechanical properties and residual stress distribution of the part were tested by tensile test and the contour method. The results show that the bulk structure is mainly columnar crystal and equiaxed crystal, and the latter layer of laser metal deposition will form a remelted zone and heat-affected zone in the former deposition zone. Tensile test results show that the tensile strength of tensile specimens parallel to laser scanning direction and perpendicular to laser scanning direction is basically the same, but the elongation of the specimens perpendicular to the laser scanning direction is relatively better. The main reason is the different distribution characteristics of columnar crystals and equiaxed crystals in the two directions. Relatively large deformation occurs on the cut surface of the specimen after low-speed wire cut. The residual stress test results indicate that tensile stress is formed in the upper part and it reaches 315 MPa at the top surface. And compressive stress is formed at the part/substrate interface and the substrate.

## 1. Introduction

316L stainless steel is known as a material with excellent corrosion resistance; it is used for various applications of the petrochemical (oil and gas) and chemical industries and as biomaterial. Additive manufacturing (AM), as an emerging manufacturing technology, has been widely used in aerospace [1, 2], medical [3, 4], construction [5], and other fields. With AM technology, a computer-aided design (CAD) model can be directly transformed into a 3D object, built layer by layer, in a relatively short time and with low cost, avoiding the long processes of conventional fabrication methods [6].

Additive manufacturing process consists of a group of modern manufacturing technologies that are used to produce three-dimensional prototypes from CAD representations [7, 8]. For example, the selective laser melting (SLM) and laser metal deposition (LMD) techniques are frequently used for stainless steel component production and for regeneration [9, 10]. Compared with SLM, LMD forming size is not limited; in ideal cases, the part size can be very large. In addition, LMD processing efficiency is relatively high; LMD has a great advantage in the case of large part size without accurate part accuracy requirement. The LMD process belongs to the category of laser cladding methods [11]. In recent years, there has been an increasing interest in the LMD process. Ma et al. obtained a well-formed additive manufacturing part using a variation-oriented raster scanning method which can improve the fabrication of precision [12]. Xiang et al. found that the elements of LMD samples were more homogeneously distributed than casting technique samples [13]. Wang et al. found that specimens perpendicular to the build direction had lower elongations than specimens parallel to the build direction [14]. However, the relation between the microstructure distribution and tensile properties is seldom discussed.

In addition, due to the complex thermal effects in the additive manufacturing process, the residual stress distribution in the part is complicated. The residual stress of the part may exceed the yield strength of the alloy, affecting corrosion resistance, fracture toughness, crack propagation behavior, and fatigue performance [15, 16]. Mukherjee et al. studied the residual stress via finite element method and predicted the part deformation after additive manufacturing [17]. Zhuo et al. carried out heat treatment to eliminate the residual stress and measured the residual stress by X-ray diffraction [18]. Bartlett et al. proposed a new method, the 3D-DIC curvature-RS model, to measure the residual stress of the part which was fabricated by additive manufacturing, and the error was only ∼6% for all measured points [19]. Szost et al. studied the residual stress distribution of thin-walled structures using neutron diffraction method which was fabricated by laser additive manufacturing and wire+arc additive manufacturing (WAAM); the results showed that higher residual stress were formed in WAAM samples [20]. In 2001, Prime first proposed the contour method to test the residual stress of components. Prime combined the finite element method with the release technique to propose the original concept of the contour test method and pointed out that this method can completely obtain the stress distribution on a section [21]. Prime et al. studied the contour test method, and the test error of this method is about 25 MPa, which can meet the test requirements of engineering and scientific research. However, few scholars use the contour method to measure the residual stress of parts manufactured by additive manufacturing.

In this paper, AISI 316L stainless steel parts were fabricated by LMD. The microstructures and tensile properties parallel to and perpendicular to the laser scanning direction were observed and tested. Fracture modes were analyzed, and residual stress distribution of the components was measured by the contour method.

## 2. Materials and Methods

**Figure 1:**
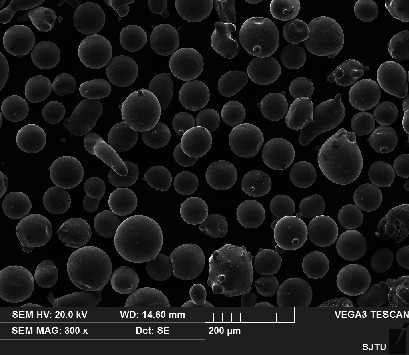
SEM picture of AISI 316L stainless steel powders.

**Figure 2:**
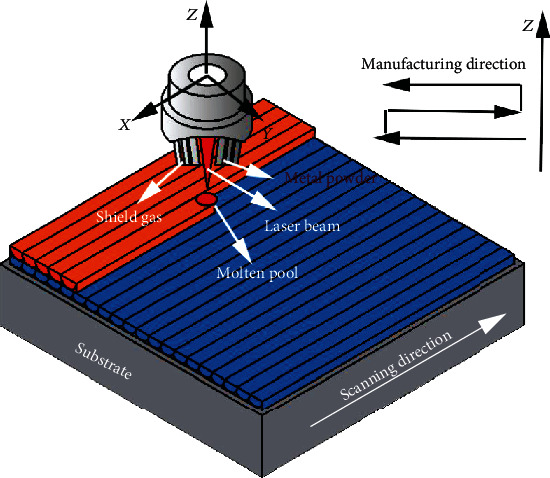
Schematic diagram of block part manufacturing.

AISI 316L alloy powder obtained by gas atomisation was used in this study. The morphology of the powder is shown in Figure 1, and the chemical composition is listed in Table 1. The powder diameter was ranging between 45 *μ*m and 75 *μ*m and had a good degree of sphericity that enabled a smooth powder feeding flow. The substrate was an AISI 1045 medium carbon steel plate with a composition listed in Table 2. The LMD experiment was carried out by using a high-power fiber laser processing system consisting of a high-power fiber laser (YLS-6000-S2T), an ABB robot arm, and a coaxial focusing powder feeding system (DPSF-2). The laser beam was modulated to a 5 mm × 5 mm square spot. The deposition was achieved at a fixed 2 kW laser power, an overlapping rate of 30%, a laser scanning speed of 4 mm/s, and a powder feeding rate of 8.8 g/min. A schematic diagram of LMD additive manufacturing process is plotted in Figure 2. The distance between the laser head and the substrate was 13 mm. This distance was consistent with the focus of the powder collection to ensure that the powder melts at the same time. When the first layer was finished, the laser head was raised by 0.8 mm in the *Z*-axis direction, which agrees with the height of each layer of the alloy. The finished alloy serves as a new substrate to carry the new alloy.

**Figure 3:**
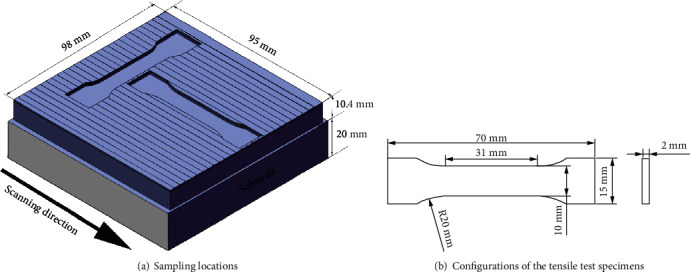
Schematic diagram of the samplings of tensile specimens.

After the completion of LMD build, the microstructures of the deposits at different locations were examined after mechanical grinding/polishing and etching by using aqua regia reagent. An optical microscope (OM) was used to observe the microstructure. It is known that an additive manufactured component exhibits anisotropic mechanical properties; therefore, in this study, tensile tests were conducted on specimens taken along both longitudinal and transverse directions.  Figure 3(a) illustrates the positions of where the tensile specimens were taken from, and Figure 3(b) shows the dimensions of the samples by following ISO 6892-1:2009 standard. The tensile tests were conducted at room temperature with a stretching speed of 2 mm/min. Each test was repeated 3 times, and an averaged ultimate tensile strength (UTS) and elongation (EL) were thereafter obtained. Finally, the fracture surface was observed by a scanning electron microscope (SEM).

**Figure 4:**
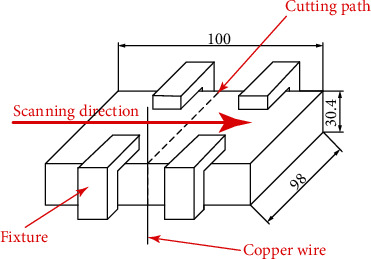
Schematic illustration of slow wire cutting process.

**Figure 5:**
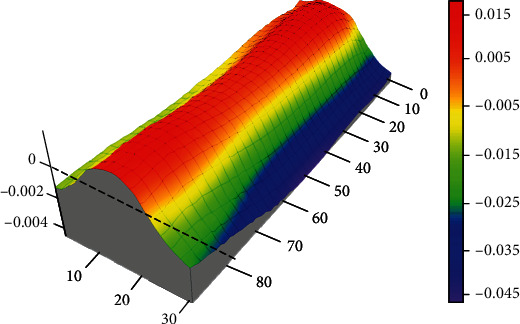
Surface contour after linear cutting (mm).

The longitudinal residual stress distribution in the LMD part was analyzed using the contour method [22, 23]. At first, the LMD block was cut into two parts along the direction perpendicular to the laser scanning direction using a slow wire cutting process as illustrated in Figure 4. The profile of the cutting surface was then deformed due to the release of residual stress. Then, the profiles of two deformed surfaces were determined by a high-precision three-axis coordinate measurement machine (CMM), and the measurement accuracy is 2 *μ*m. The acquired contour point clouds from two opposite surfaces were subsequently processed by averaging and bivariate spline fitting to generate a smooth surface and eliminate measurement errors. The representative profile of deformed cutting surface is shown in Figure 5. Finally, the measured contour was used as the boundary condition of the finite element model. The elastic state is used to analyze the stress state of the deformed cutting surface after returning to the plane state before cutting (or changing the cutting surface in the planar state to the deformation contour in the opposite direction). It is assumed that no plastic deformation occurs, and the obtained stress on the cutting surface is equivalent to the original stress at the same position before the cutting.

## 3. Results and Discussion

### 3.1. Microstructure

**Figure 6:**
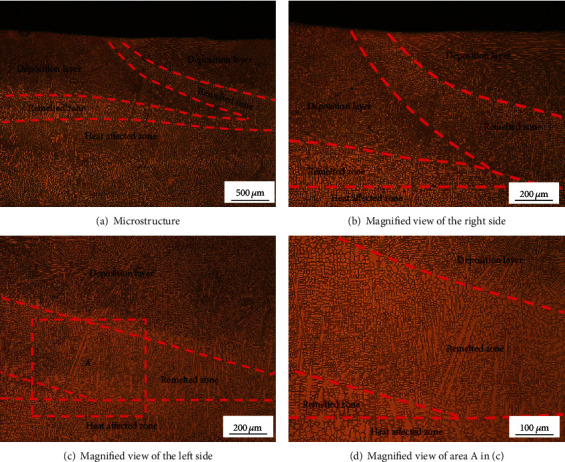
Cross-sectional microstructure perpendicular to the laser scanning direction.

Figure 6 shows the microstructure of the cross section perpendicular to the laser scanning direction.  Figure 6(a) indicates that the microstructure of the LMD part consists of a deposition layer zone, remelted zone, and heat-affected zone due to the laser heat input during the latter deposition process. Figures 6(b) and 6(c) indicates that the microstructure of the remelted zone grew from the heat-affected zone of the former layer. The direction of microstructure growth depends on the direction of the structure in the heat-affected zone. In the center zone of the remelted zone, the direction of microstructure growth is no longer the same. This is due to the fact that the remelted portion has unmelted grains which become the nucleation, resulting in a nonuniform growth direction of the microstructure. In addition, it can be seen that the microstructure of the latter deposited layer is fine, the upper part is equiaxed crystals, and the lower part is fine columnar dendrites. In area A (Figure 6(d)), the microstructure growth direction of this region is consistent, and the growth direction is along the direction with the largest temperature gradient. It can be seen that the microstructure is relatively coarse and some columnar dendrites are formed in it.

**Figure 7:**
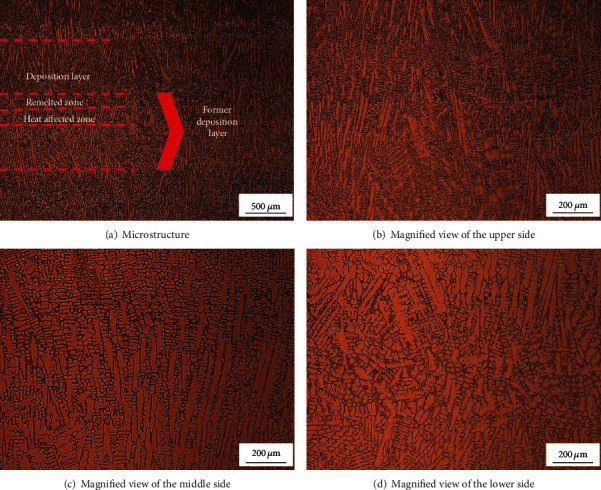
Cross-sectional microstructure parallel to the laser scanning direction.

Figure 7 shows the microstructure of the cross section parallel to the laser scanning direction. The latter deposition layer zone, remelted zone, and heat-affected zone are also observed in the LMD part. It can be seen that the microstructure in this direction is mainly composed of columnar dendrites. The growth direction of the columnar dendrites is not uniform. This is due to the complicated thermal process of the LMD manufacturing process, which causes the temperature gradient direction of the microstructure to change greatly during the growth, thus causing the growth direction of the columnar crystal to be inconsistent when the part is finally completed. Since the initial cooling rate is low, heterogeneous nucleation starts on the unmelted crystal grains, and relatively coarse columnar crystals are formed, and then, the cooling rate is increased, and the columnar crystals start to grow in a direction in which the temperature gradient is large on the basis of the coarse columnar crystal. As can be seen in Figure 7(b), the direction of microstructure growth in the remelted portion is chaotic, and some columnar crystals grow across the heat-affected zone and the remelted zone.  Figure 7(c) indicates that there are columnar crystals before the remelting process. New grains grow from the unmelted short columnar crystals.  Figure 7(d) shows that the heat-affected zone is mainly coarse columnar dendrites and the growth direction of the dendrites is basically the same.

### 3.2. Tensile Properties

**Figure 8:**
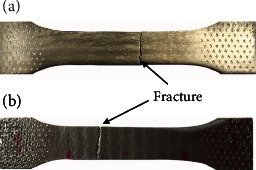
Photos of the specimens after tensile tests: (a) perpendicular to laser scanning direction; (b) parallel to laser scanning direction.

**Figure 9:**
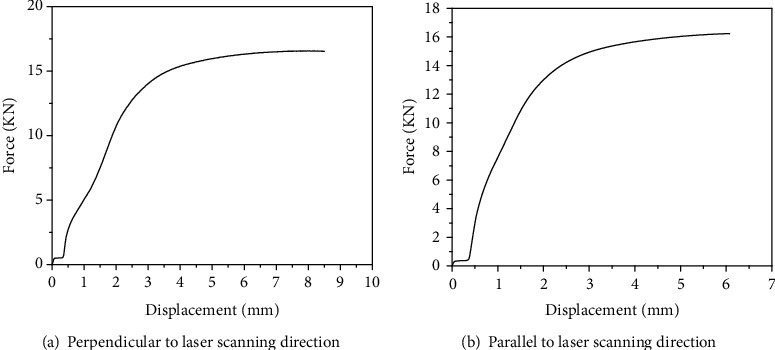
Force-displacement curve of the tensile test specimens: (a) perpendicular to laser scanning direction; (b) parallel to laser scanning direction.

Figure 8 shows the photographs of the samples after tensile test. Figure 8(a) is a specimen perpendicular to the laser scanning direction, and Figure 8(b) is a specimen parallel to the laser scanning direction. Figure 8 shows that no “necking” phenomenon happened at the fracture position during the tensile test process. Because the local strains in the neck are large, necking is often closely associated with yielding, a form of plastic deformation associated with ductile materials. The neck eventually becomes a fracture when enough strain is applied. It indicates that the ductility of the 316L LMD part fabricated in this study is not fine.  Figure 9 is the force-displacement curve of the tensile specimen obtained in two directions, and it can also be seen from Figure 9 that there was no yielding that happened, and the specimens fractured directly in the tensile test process.  Table 3 shows the tensile properties of the two kinds of specimens.  Table 3 indicates that the tensile strengths in the two directions are basically the same, about 800 MPa. However, the elongation of the specimen perpendicular to the laser scanning direction is 3.0% larger than that of the specimen parallel to the laser scanning direction. According to the OM results of the LMD part (Figures 6 and 7), a large number of coarse dendrites are found to distribute along the direction parallel to the laser scanning direction, which may decline the mechanical properties of the part. As a result, the elongation of the specimens parallel to the laser scanning direction was only 7.3%. In contrast, the specimen perpendicular to the laser scanning direction displayed higher elongation.

**Figure 10:**
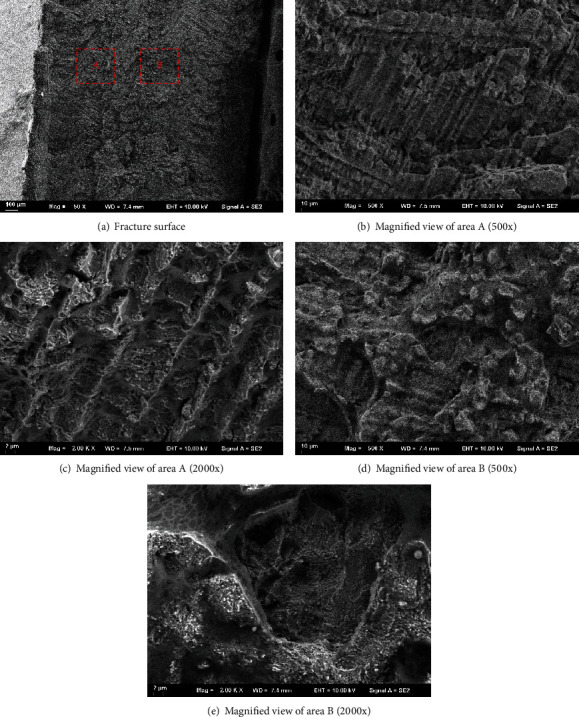
SEM photos of the fracture surface.

Figure 10 shows the SEM photos of the tensile fracture surface for specimen perpendicular to the laser scanning direction. It can be seen from the macroscopic morphology that the fracture surface of the two regions A and B is different. There is a herringbone pattern in region A. Further enlarged photos of regions A and B are shown in Figures 10(b)–10(e). It can be seen that the cleavage plane can be clearly seen in region A. The fracture mode is the cleavage fracture. The fracture mode is a quasicleavage fracture in region B. Because of the presence of fine microstructures in region B, the quasicleavage facet is not a crystallographic cleavage plane, which is quite different from region A.

**Figure 11:**
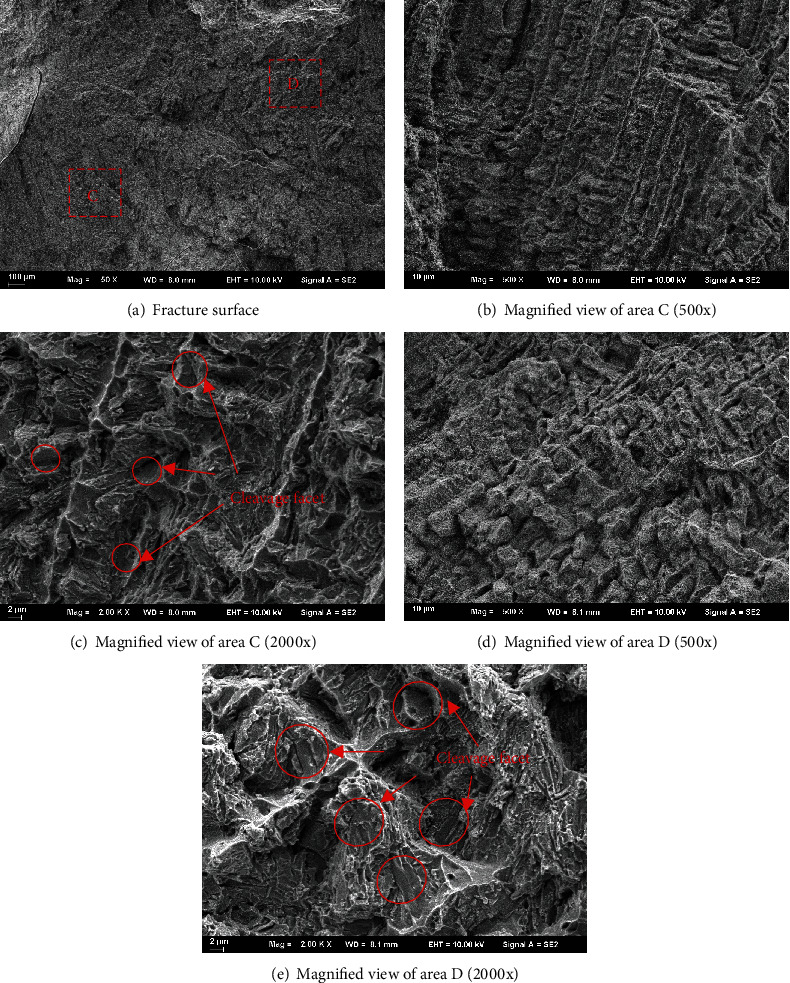
SEM photos of the fracture surfaces in parallel to the laser scanning direction.

Figure 11 shows the SEM photos of the tensile fracture surface for the specimen parallel to the laser scanning direction. It is very different comparing with the fracture morphology of specimens perpendicular to the laser scanning direction in Figure 10. The fracture mode of the tensile specimen is the cleavage fracture. The cleavage facets can easily be seen in Figures 11(b)–11(e), magnified view of regions C and D in Figure 10(a). This is also due to the formation of coarse dendrites in this direction. And the results agree well with the differences of elongation ratio for specimens at two directions.

### 3.3. Residual Stress Distribution

**Figure 12:**
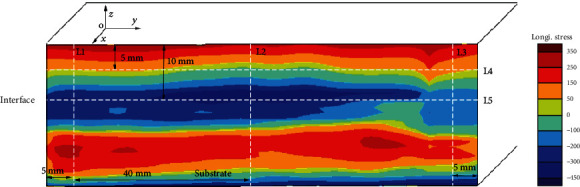
Longitudinal residual stress distribution in the LMD part (MPa).

Figure 12 shows the distribution of longitudinal residual stress after reconstruction, and it can be seen that along the thickness direction, the top region is tensile stress and the middle region is compressive stress. The compressive stress near the interface can reach up to -375 MPa, and the tensile stress in the top region is the largest, and the maximum is 320 MPa. Because additive manufacturing will be accompanied by remelting and the cooling rate of this area is slow, the contraction of this area is stretched by the previous layer of metal, so tensile stress of cracks will appear. Through the progress of the additive manufacturing process, tensile stress will be concentrated in the center zone. With the progress of additive manufacturing, the longitudinal residual stress gradually increases with the increase of the number of layers, but because the heat effect of the latter layer on the previous layer is equivalent to the heat treatment of the previous layer, the stress increases slowed down.

**Figure 13:**
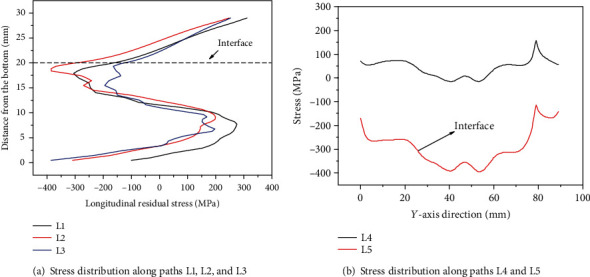
Stress distribution along the five typical paths in Figure 12.

Figure 13 shows the residual stress line distribution along five typical paths (L1 to L5 in Figure 12). It can be seen from Figure 13(a) that the variation of the three paths (L1, L2, and L3) is almost the same, from the top to the interface; the tensile stress is gradually changed to the compressive stress; and the maximum tensile stress reaches 315 MPa. In the laser metal deposition area, the paths L1 and L3 on both sides of the part are more similar, and the areas on both sides are first changed from compressive stress to tensile stress. This is because the areas on both sides of the part can be freely deformed parallel to the scanning direction during the LMD process. In the process of LMD, as the deformation of the part releases the stress, the stress in the two sides changes from compressive stress to tensile stress, and the intermediate zone is restrained by the constraints of the block parts on both sides. The process of changing to tensile stress slows down.  Figure 13(b) indicates that the variation of the stress distribution along the paths L4 and L5 (interface) is completely opposite. The small sudden change on the right side of L4 and L5 are due to the oscillation of copper wire during the low-speed wire cut process, making the profile inaccurate, and the actual stress does not mutate. At the same position on the *Y*-axis, when the compressive stress on the path L5 is the largest, the tensile stress on the path L4 is the smallest. Along the path L4, the tensile stress on both sides is the largest, about 100 MPa, and gradually decreases toward the middle, and it is compressive stress in the middle portion. The compressive stress on both sides of the path L5 is the smallest, about -180 MPa, and the compressive stress in the middle region is the largest, which can reach -375 MPa.

## 4. Conclusions

The microstructure results show that the LMD 316L parts are mainly composed of columnar crystals and equiaxed crystals. The microstructure distributions perpendicular and parallel to the laser scanning direction are different, resulting in differences of tensile properties in the two directions. The sample elongation of the specimens perpendicular to the laser scanning direction is 3% higher than that in the parallel direction, and the tensile strengths in the two directions are almost the same. The contour stress measurement results show that the tensile stress is observed at the upper side and then changes gradually to compressive stress at the bottom in the LMD part. The maximum tensile stress and compressive stress are 320 MPa and -375 MPa, respectively.

## Figures and Tables

**Table 1:** Chemical compositions of AISI 316L stainless steel powder (wt. %).

Element	C	Si	Cr	Ni	S	Fe
Content	<0.08	0.76	17.41	12.04	0.60	Bal.

**Table 2:** Chemical compositions of AISI 1045 substrate (wt. %).

Element	C	Si	Mn	Cr	Ni	Cu	Fe
Content	0.42~0.50	0.17~0.37	0.50~0.80	0.25	0.30	0.25	Bal.

**Table 3:** Tensile test results of the 316L LMD part.

Direction	UTS (MPa)	Average value (MPa)	EL	Average value
Perpendicular to laser scanning direction	804	804.3	9%	10.3%
	800		10%	
	809		12%	

Parallel to laser scanning direction	791	791.7	7%	7.3%
	799		8%	
	785		7%	

## Data Availability

The data used to support the findings of this study are available from the corresponding author upon request.
